# Insights into the immunological description of cryoglobulins with regard to detection and characterization in Slovenian rheumatological patients

**DOI:** 10.1007/s12026-023-09434-9

**Published:** 2023-11-23

**Authors:** Manca Ogrič, Tinka Švec, Katjuša Mrak Poljšak, Katja Lakota, Eva Podovšovnik, Marie Nathalie Kolopp-Sarda, Alojzija Hočevar, Saša Čučnik

**Affiliations:** 1 Department of Rheumatology, University Medical Centre Ljubljana, Ljubljana, Slovenia; 2 FAMNIT, University of Primorska, Koper, Slovenia; 3 Valdoltra Orthopaedic Hospital, Ankaran, Slovenia; 4 Immunogenomics and Inflammation Research, University of Lyon, Lyon, France; 5 Immunology Laboratory, University Hospital Lyon, Lyon, France; 6 Faculty of Medicine, University of Ljubljana, Ljubljana, Slovenia; 7 Faculty of Pharmacy, University of Ljubljana, Ljubljana, Slovenia

**Keywords:** Cryoglobulins, Cryoglobulinemic vasculitis, Rheumatoid factor activity, Complement components, Cryoglobulin detection, Pre-analytical phase

## Abstract

The detection of cryoglobulins (CG) used to diagnose cryoglobulinemic vasculitis requires strict adherence to protocol, with emphasis on the preanalytical part. Our main objectives were to introduce a more sensitive and specific protocol for the detection of CG and to characterize CG in Slovenian patients diagnosed with cryoglobulinemic vasculitis, other vasculitides, connective tissue diseases or non-rheumatic diseases examined at the Department of Rheumatology (University Medical Centre Ljubljana). Samples were routinely analyzed for the presence of CG with the protocol using the Folin-Ciocalteu reagent. In the newly introduced protocol, the type of CG was determined by immunofixation on visually observed positive samples and the concentration of CG in the cryoprecipitate and rheumatoid factor (RF) activity were measured by nephelometry. RF, C3c and C4 were measured in patients` serum and a decision tree analysis was performed using all results. The agreement between negative and positive results between the two protocols was 86%. Of the 258 patient samples tested, we found 56 patients (21.7%) with positive CG (37.5% - type II, 62.5% - type III). The RF activity was observed in 21.4% of CG positive subjects. The median concentration of type II CG was significantly higher than that of type III CG (67.4 mg/L vs. 45.0 mg/L, p = 0.037). Patients with type II had lower C4 concentrations and higher RF compared to patients with type III CG. In the decision tree, C4 was the strongest predictor of cryoglobulinemia in patients. With the newly implemented protocol, we were able to improve the detection and quantification of CG in the samples of our rheumatology patients and report the results to adequately support clinicians.

## Introduction

Cryoglobulins (CG) are serum immunoglobulins that precipitate in vitro at temperatures below 37 °C, usually at 0–4 °C, and re-dissolve upon rewarming [1]. The term “cryoglobulin” was first used by Lerner and Watson in 1947 [2], but CG were observed earlier, in 1933, when Wintrobe and Buell observed abnormal precipitation of proteins on cooling serum from a patient with multiple myeloma [3]. Two proteins of size 19 S and 7 S associated by an antibody-antigen mechanism were described in a cryoprecipitate by LoSpalluto et al. [4], which were later found to be IgM with rheumatoid factor (RF) activity associated with IgG [5, 6]. The phenomenon of cold insolubility of CG is not fully understood, but it could be related to amino acid composition (mutation, insertion or deletion) or atypical glycosylation, although it mostly depends on low temperatures causing steric modifications that trigger cryoprecipitation [7, 8].

The presence of CG – i.e., cryoglobulinemia - may be transient (e.g., after acute infection) or persistent and asymptomatic, or may lead to symptoms and signs of immune complex vasculitis. High CG concentrations may cause hyperviscosity. Cryoglobulinemic vasculitis typically affects the skin, peripheral nerves, joints, and kidneys. Cryoglobulinemic vasculitis is rarely idiopathic and often occurs in association with various chronic inflammatory diseases and chronic infections [9–14].

Brouet et al. proposed a classification of CG into types I to III, which is widely used because of its correlation with the clinical presentation. The classification is based on the immunochemical properties of CG and indicates the cause of CG occurrence [15].

Type I CG comprises a single type of monoclonal immunoglobulin, usually of IgM or IgG isotype, whose production is related to the underlying lymphoproliferative disease (e.g., multiple myeloma, monoclonal gammopathy of unknown significance, B-cell disorders such as Waldenström macroglobulinemia, and lymphoproliferative disorders such as non-Hodgkin’s lymphoma and chronic lymphocytic leukaemia) [16, 17]. Type II CG consists of both monoclonal and polyclonal immunoglobulins, with the most common CG consisting of monoclonal IgM with RF activity and polyclonal IgG. Type III CG consists of polyclonal immunoglobulins, most commonly polyclonal IgM with RF activity and polyclonal IgG. The latter two types are referred to as mixed CG and are often associated with infections (most commonly hepatitis C) and autoimmune diseases [17–19]. Of the cryoglobulinemias, 10–15% are type I, 50–60% are type II, and 30–40% are type III. In addition to immunoglobulins, other proteins may be found in the cryoprecipitate (e.g., albumin, fibronectin, fibrinogen, viruses, and bacteria) [20]. RF alone does not precipitate in the cold, but it does if it is bound to polyclonal immunoglobulins directed against the stimulating agents (viruses, bacteria, or specific immunogens) [9]. Hypocomplementemia is often observed in association with cryoglobulinemia and is manifested by decreased C1q, C2, and C4, normal or below threshold C3c, and decreased or below threshold CH50 [21].

Appropriate laboratory detection of CG involves simple biochemical quantification of the precipitate. Detection of CG is performed from laboratory to laboratory using different analytical approaches, resulting in heterogeneity and non-comparable results. CG results can only be accurate if strict pre-analytical conditions at 37 °C are maintained to avoid precipitation of CG during the coagulation process, which helps to avoid false-negative test results [22–24]. Another reason for false-negative results could be an insufficient volume of serum, because the precipitate is not visible. Therefore, the volume of serum should be at least 3–5 mL. Some CG need calcium for precipitation, so tubes without calcium chelators such as EDTA should be used [25]. However, false positive results may also occur in samples from patients receiving heparin therapy, as heparin could form a complex with fibronectin, which is also a cold-insoluble protein, or in samples from patients with dyslipidemia, as lipids also form visible precipitates [26]. False positivity due to lipids was successfully prevented as precipitates were observed at the top of the serum and not at the bottom as cryoprecipitation. The international standardization is still needed. However, several approaches have been described and proposed as a first step toward standardization of CG detection.

Our objectives were (a) to introduce a new protocol (more sensitive and specific according to data) for the detection of CG according to Kolopp-Sarda et al. [27], (b) to compare this protocol with the detection of CG with the Folin-Ciocalteu reagent (FC), which has been used in our laboratory for decades, (c) to characterize CG in the Slovenian patients examined at the Department of Rheumatology, University Medical Centre Ljubljana, and (d) to determine potential predictors of cryoglobulinemia.

## Methods

### Patients

Serum samples were collected from October 2022 to May 2023 from patients with known or newly diagnosed cryoglobulinemic vasculitis, other vasculitides, connective tissue diseases, and from patients without a diagnosis of rheumatic disease who were sent for routine CG testing and analyzed at the Immunology Laboratory of the Department of Rheumatology, University Medical Centre Ljubljana, Slovenia. The study was conducted according to the guidelines of the Declaration of Helsinki and approved by the National Medical Ethics Committee, Ljubljana, Slovenia (0120–133/2022/4).

### Folin-Ciocalteu (FC) protocol

- **Sampling**

Samples were collected in pre-warmed 4 mL tubes without gel or anticoagulant at 37 °C, transported in a pre-warmed thermostable transport box, and kept in the incubator at 37 °C for a two-hour clotting period. Blood samples were centrifuged three times for 10 min at 37 °C (1800 g). Sera were transferred to conical-bottomed test tubes (3 tubes of 600 μL each) and stored at 4 °C.

- **Isolation of CG**

After 5–8 days of incubation at 4 °C, the cryoprecipitate was separated by cold centrifugation (30 min, 1800 g) and washed three times in 2 mL cold PBS. After the last wash, samples were treated with 600 μL PBS and placed in an incubator at 37 °C for up to one hour to dissolve the precipitate.

- **Quantification of CG**

The presence of proteins from the dissolved cryoprecipitate was confirmed using Folin-Ciocalteu (FC) reagent. In the first step, 3 mL of reagent D (2%Na2CO3 in 0.1 M NaOH, 1% CuSO4 × 5 H2O, 2% K, Na tartrate) was added to 200 μL of the sample and incubated for 10 min. Then 600 μL of the reagent FC was added and after 30 min of incubation, the absorbance was measured at 720 nm. A concentration greater than 100 mg/L was considered a positive result, while clinically significant concentrations were defined as concentrations greater than 300 mg/L [28]. At concentrations greater than 100 mg/L, CG isotypes (IgG, IgM, IgA) were determined from stored sera by radial immunodiffusion according to the manufacturer`s instructions (Siemens Healthcare).

### Protocol by Kolopp-Sarda et al

According to Kolopp-Sarda et al. [27], we introduced the new protocol for detection and characterization of CG – with sampling, visual observation, isolation of CG, classification of CG, quantification of CG and in the last step additional analyses of the activity of RF in cryoprecipitate and serum.

- **Sampling**

**Fig. 1:**
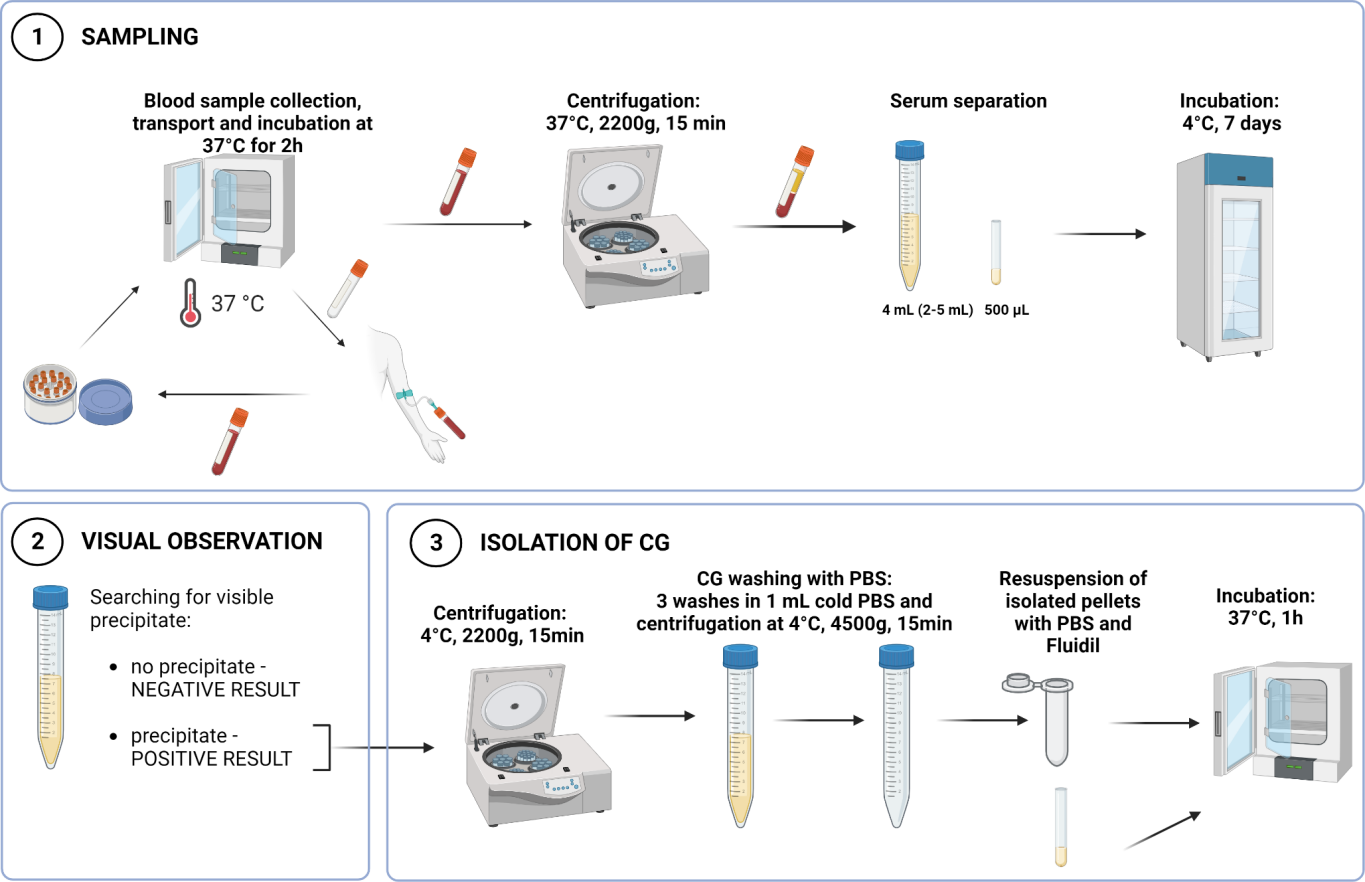
Graphical representation of sampling, visual observation and isolation of CG. Legend: CG – cryoglobulins, PBS – phosphate buffered saline. The figure was created with BioRender

Samples were collected in pre-warmed 4 mL-tubes without gel or anticoagulant at 37 °C, transported in pre-warmed thermostable transport box and kept in the incubator at 37 °C for a two-hour clotting period. After the two-hour clotting time at 37 °C, blood samples were centrifuged at the same temperature (2200 g). The sera were transferred to conical-bottomed test tubes and stored at 4 °C for seven days (Fig. 1-1).

- **Visual observation**

**Fig. 2:**
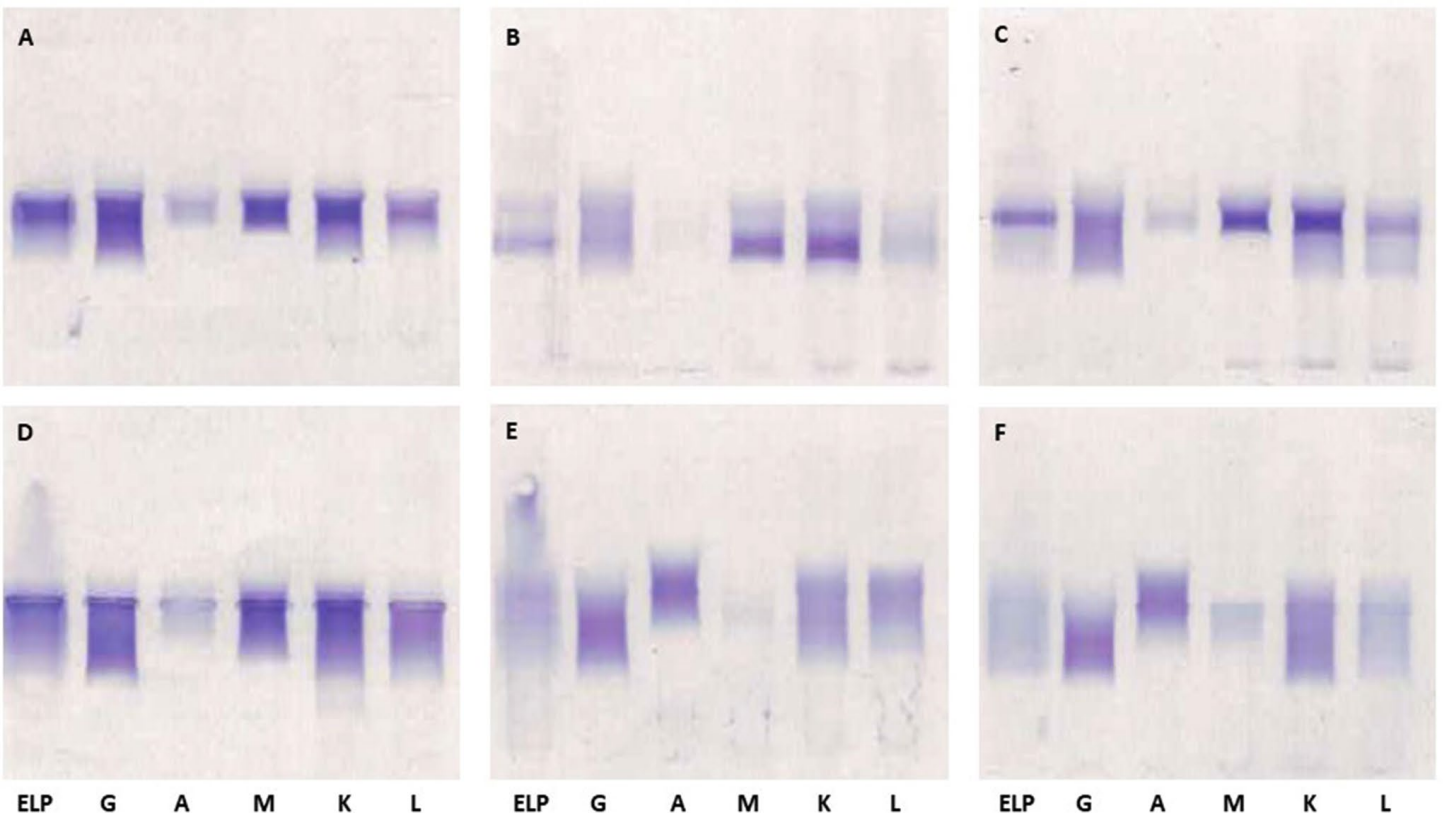
Examples of immunofixation gels used to determine cryoglobulin type (**A-C**: Type II CG, polyclonal IgG and monoclonal IgM kappa, **D-F**: Type III CG, polyclonal IgG and IgM (D) and polyclonal IgG and IgA (E-F))

Visual analysis of serum was performed after incubation at 4 °C for 7 days (Fig. 1-2). Samples with visible precipitate were further analyzed, and others were considered negative.

- **Isolation of CG**

**Fig. 3:**
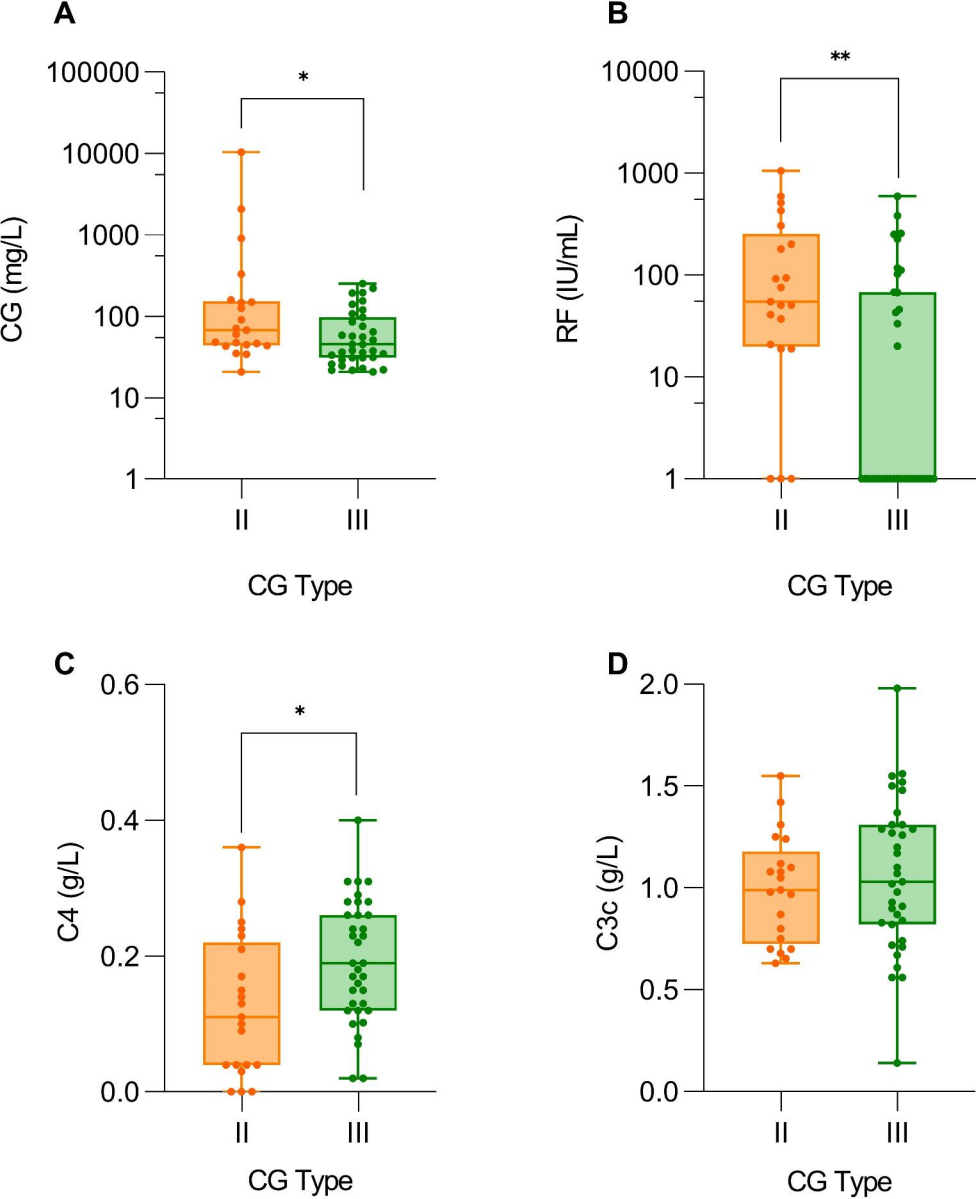
Comparison between CG concentrations (**A**), RF (**B**), C4 (**C**) and C3c (**D**) in serum between the patients with type II or type III CG. Legend: CG – cryoglobulins, RF – rheumatoid factor. Asterisks are used as follows: * for p < 0.05 and ** for p < 0.001

The volume of serum supernatant was carefully measured to calculate the final concentration of CG. Cold centrifugation (15 min, 2200 g, 4 °C) was performed to isolate CG. The cryoprecipitate was then purified in 2-mL tubes by washing three times in 1 mL of cold PBS. After each wash, samples were centrifuged at 4500 g for 15 min. After the final wash, 500 μL of PBS with Fluidil® (2%, Sebia, Lisses, France) was added to the cryoprecipitate and placed in an incubator at 37 °C for one hour to dissolve the precipitate (Fig. 1-3).

- **Classification of CG**

Immunofixation of dissolved isolated CG was performed according to the manufacturer`s instructions (Hydragel 4IF, Sebia, Lisses, France) to determine the type of CG, isotypes (IgG, IgA, or IgM; kappa or lambda), monoclonality or polyclonality (Fig. 2). Samples were first applied to an agarose gel and then CG were separated by electrophoresis. The sample is simultaneously electrophoresed in six lanes to find and identify the CG. Then antisera were applied to the gel and the process of immunofixation began. One lane is used as a reference to show the overall electrophoretic pattern of the isolated proteins in the sample after electrophoresis. The remaining five lanes allow identification of the isotype of CG - gamma (Ig G), alpha (Ig A) and mu (Ig M) heavy chains and kappa and lambda light chains. When the appropriate immunoglobulins are present, the antisera diffuse into the gel and precipitate them. Blotting and washing of the gel removes the soluble, non-precipitated immunoglobulins. The gel matrix contains the precipitin of the antigen-antibody combination and staining with acid blue was used to identify the precipitated CG.

- **Quantification of CG**

After identification of immunoglobulin isotypes by immunofixation, the concentration of isolated immunoglobulins was determined with the Atellica NEPH 630 nephelometer (Siemens, Heatlhineers, Erlangen, Germany) using reagents for low Ig concentrations. The final concentration of CG is adjusted to the initial serum volume and expressed in mg/L. A threshold CG concentration of 30 mg/L was defined as a clinically important value [29], whereas concentrations between 20 and 30 mg/L were defined as a grey zone.

- **Analyses of RF**

For positive samples, RF activity (not isotype specific) was measured in cryoprecipitate using the Atellica NEPH 630 nephelometer and, if positive, also in serum at 37 °C.

- **Verification**

For immunofixation, we determined analytical sensitivity, between-run precision, and within-run precision for immunoglobulin detection in CG. Serial dilutions of samples were prepared and analyzed on the gel. The last visible dilution was defined as the analytical sensitivity after calculating the concentration from the concentration measured with nephelometer. For between-run precision, a commercial control was used and tested on three different gel lots on different days, whereas for within-run precision, a patient sample was used and tested 4 times on one gel.

To determine the precision of IgG, IgM, and IgA concentration by immunonephelometry, a commercial control was used and within- and between-run precision was calculated. Control samples were tested 5 times per day for 5 days to obtain 25 results for each sample.

### Additional analysis of serum

RF and the concentrations of complement fractions C3c and C4 were measured in the serum of all patients using an Atellica NEPH 630 nephelometer (Siemens Heatlhineers, Erlangen, Germany).

### Statistical analysis

The overall agreement between the results of the two protocols was calculated. Spearman’s rank correlation coefficient was used to analyze quantitative correlation and Cohen’s kappa test was used to determine qualitative agreement between the results. The Mann-Whitney U test and Kruskal-Wallis test were used to compare groups because of the nonnormal distribution of the data. P values of less than 0.05 were considered significant. Data are presented as median (25th -75th percentile). Statistical analyses were performed using Analyse-IT for Microsoft Excel (Analyse-IT Software Ltd., Leeds, UK).

The CHAID decision tree was calculated to find predictors (SPSS IBM). Specifically, the overall accuracy of the CHAID decision tree model for predicting cryoglobulinemia and the percentage of patients correctly classified as negative or positive were calculated. Using these results, the index of Gains for nodes was calculated as the ratio of the response percentage for the target category compared to the response percentage for the entire sample. An index greater than 100% indicated significant prediction accuracy.

## Results

### Patients

Our study cohort consisted of 258 patients (median age 63 (47–72) years, 194 women (75.2%)) from the Department of Rheumatology, University Medical Centre Ljubljana with a diagnosis of cryoglobulinemic vasculitis (n = 18, 7.0%), other vasculitides (n = 55, 21.3%), other connective tissue diseases (n = 130, 50.4%) and other non-rheumatic diseases (n = 55, 21.3%) (Table 1). Samples were collected from patients at the time of diagnosis or at follow-up, so some patients had already been treated. The cryoglobulinemic vasculitis group consisted of 18 patients, 8 of whom received no therapy, 3 DMARDs only, 3 glucocorticoids only, and 4 glucocorticoids and DMARDs.

**Table 1:** Characteristics of the included patients

All patients	
Number	258
Sex ration (M/F)	64/194
Age, median, IQR (years)	63 (47–72)
**Groups of patients**	
**Cryoglobulinemic vasculitis, number (% of all)**	**18 (7.0)**
*Primary*	9
*Secondary to Sjögren`s syndrome*	7
*Secondary to virus infection*	2
**Other vasculitides, number (% of all)**	**55 (21.3)**
*IgA vasculitis*	31
*Giant cell arteritis*	10
*ANCA-associated vasculitis*	9
*Secondary vasculitis*	1
*Skin limited vasculitis*	2
*Polyarteritis nodosa*	1
*Microvascular vasculitis*	1
**Connective tissue diseases, number (% of all)**	**130 (50.4)**
*Sjögren`s syndrome*	62
*Rheumatoid arthritis(RA)*	6
*Antiphospholipid syndrome (APS)*	6
*Polymyalgia rheumatica*	7
*Systemic lupus erythematosus (SLE)*	1
*Systemic sclerosis (SSc)*	7
*Sjögren`s syndrome and SLE/RA/SSc/myositis/lymphoma/lupus/APS*	24
*Spondiloartritis*	2
*Psoriasis*	1
*Mixed connective tissue disease*	1
*Other*	13
**Non-rheumatic diseases, number (% of all)**	**55 (21.3)**
*Raynaud`s disease*	13
*Urticaria*	4
*Fibromyalgia*	4
*Ischemic lesions of CNS*	4
*Other*	30

### The results of Kolopp-Sarda et al. protocol and comparison to FC protocol

Between October 2022 and May 2023, both methods for CG detection (FC protocol and Kolopp-Sarda et al. protocol) were performed in parallel. Using the clinically important threshold of 300 mg/L with the FC protocol [28] and 20 mg/L with the introduced protocol (adopted from Vermeersch et al. [29]: 20–30 mg/L – positive – grey zone, > 30 mg/L clinically important threshold), the agreement between negative and positive results was 86.0% and Cohen`s kappa 0.483 (95% CI 0.346–0.619), representing only moderate agreement between the two protocols. The results of samples positive in both assays (> 300 mg/L in the FC protocol and > 20 mg/L in the Kolopp-Sarda protocol) were used to determine the correlation. The Spearman correlation coefficient was 0.692 (95% CI 0.370–0.865, p = 0.0003), indicating a moderately positive correlation (Table 2).

**Table 2:** Agreement and correlation between the results

n = 258		Kolopp-Sarda et al. protocol, concentration of CG (mg/L)	
		< 20	> 20
FC protocol, concentration of CG (mg/L)	< 300	200	34
	> 300	2	22
Agreement (%)		86.0	
Cohen`s kappa (95% CI)		0.483 (0.346–0.619)	
Spearman correlation coefficient (95%CI, p)		0.692 (0.370–0.865, 0.0003)	

### Implementation of the Kolopp-Sarda et al. protocol – results of the verification process

We determined the analytical sensitivity for the most common immunoglobulins detected by immunofixation. The analytical sensitivity for polyclonal IgG was 18 mg/L, whereas it was 21 mg/L for monoclonal IgM. We also determined the analytical sensitivity for polyclonal IgA, which was 19 mg/L. Considering serum dilution, the sensitivity with respect to the final serum concentration was 2.23 mg/L for IgG, 2.62 mg/L for IgM, and 2.34 mg/L for IgA.

We used a commercial control to determine the between-run precision of immunofixation. The control serum (containing IgG, IgM, and IgA) was tested with three different lot numbers on three different days, and we obtained identical immunofixation results, polyclonal IgG, IgM, and IgA. Similarly, identical results were obtained when we tested a sample of patients containing polyclonal IgG and monoclonal IgM kappa 4 times on one gel. Thus, we confirmed the within-run precision of immunofixation.

Precision for nephelometric detection of IgG, IgM, and IgA was determined using commercial controls. Within-run precision was 2.9% for IgG, 2.0% for IgM, and 3.3% for IgA, and between-run precision was 3.4% for IgG, 2.2% for IgM, and 3.3% for IgA.

### The presence of CG in patients and their relation to level of C3c, C4, and RF in serum

Using the Kolopp-Sarda et al. protocol, 56 out of 258 samples tested (21.7%) had total immunoglobulin concentrations greater than 20 mg/L, of which 21/56 (37.5%) were type II CG and 35/56 (62.5%) were type III CG. RF activity was detected in 8/21 (38.1%) type II CG, and in 4/35 (11.4%) samples with type III CG. Overall, RF activity was observed in 12/56 (21.4%) samples.

In patients with type II CG the most common combination of immunoglobulins, polyclonal IgG and monoclonal IgM kappa, was present in 16/21 (76.2%). Other combinations found were polyclonal IgG and IgA with monoclonal IgM kappa (n = 4; 19.0%) and polyclonal IgG and IgM and monoclonal IgA lambda (n = 1, 4.8%).

Of the 35 patients who had type III CG, 17/35 (48.6%) samples were associated with polyclonal IgG and IgM, 8/35 (22.9%) with polyclonal IgG, IgM, and IgA, 7/35 (20.0%) with polyclonal IgM only, 2/35 (5.7%) with polyclonal IgG only, and 1/35 (2.9%) with polyclonal IgG and IgA.

The median concentration of type II CG ((67.4 (44.2–152.15) mg/L, range 20.7–10417.5 mg/L) was significantly higher than that of type III CG ((45.0 (30.8–95.1) mg/L, range 20.6-253.8 mg/L), (p = 0.037) (Fig. 3A). In addition, patients with type II CG had higher RF (Fig. 3B) and lower C4 concentrations in serum (Fig. 3C) compared to patients with type III CG, while there were no significant differences in C3c in serum (Fig. 3D).

**Fig. 4:**
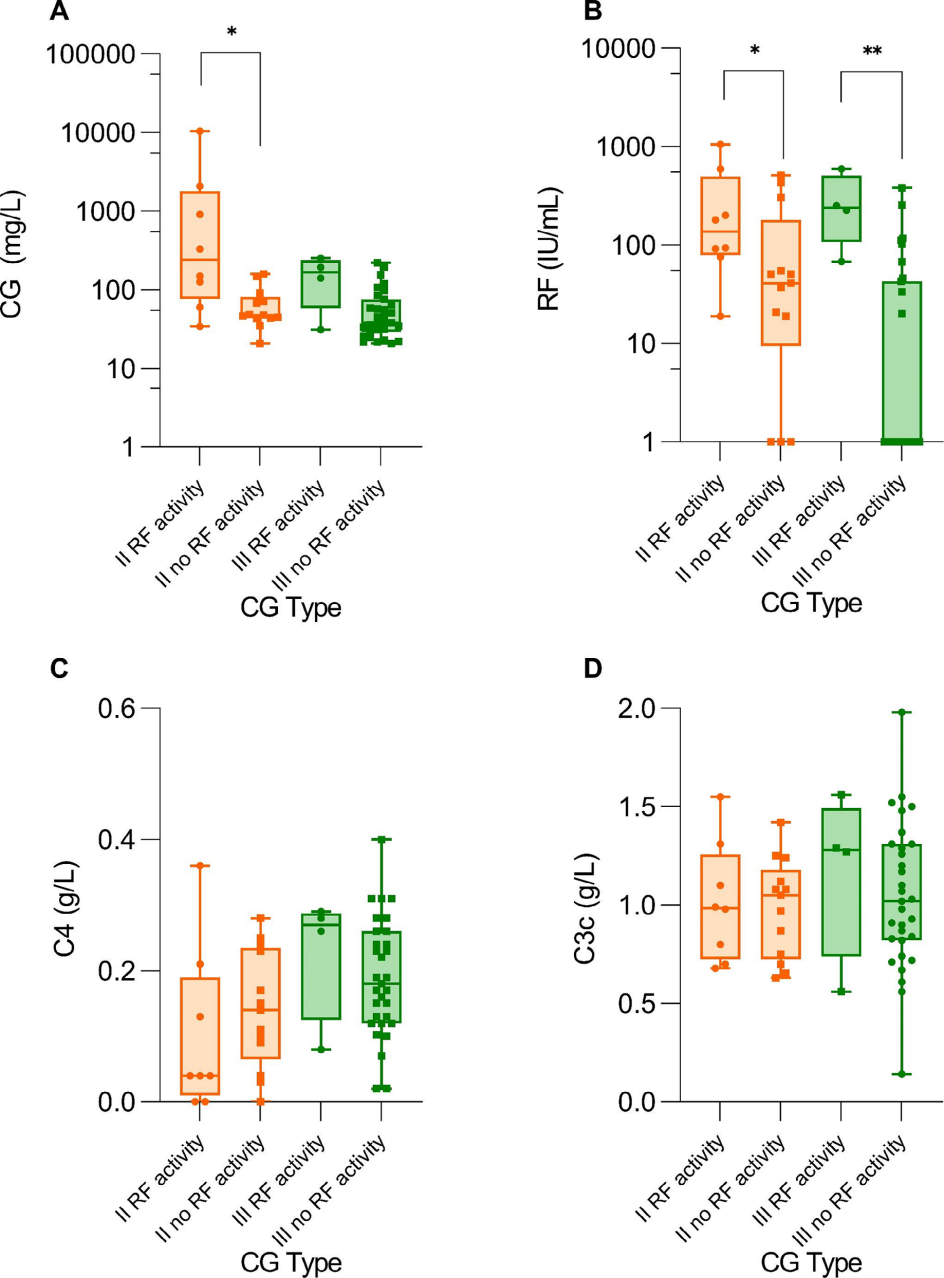
Comparison between CG concentrations (**A**), RF (**B**), C4 (**C**) and C3c (**D**) in serum between the patients with type II CG with or without RF activity in CG precipitate or type III CG with or without RF activity in CG precipitate Legend: CG – cryoglobulins, RF – rheumatoid factor. Asterisks are used as follows: * for p < 0.05 and ** for p < 0.001

In addition, patients with type II CG with RF activity had higher levels of CG compared to patients without RF activity (239.22 (87.00–1952.16) mg/L) vs. 47.63 (43.45–77.43) mg/L, p = 0.020) (Fig. 4A) and also higher levels of RF in serum (p = 0.045) (Fig. 4B). No differences in C3c and C4 concentrations were observed in these patients (Fig. 4C-D). The concentrations of CG did not differ in the group of type III CG patients with or without RF activity (p = 0.0779), but the RF in serum did (p = 0.003) (Fig. 4A-B).

### Potential predictors of cryoglobulinemia

**Fig. 5:**
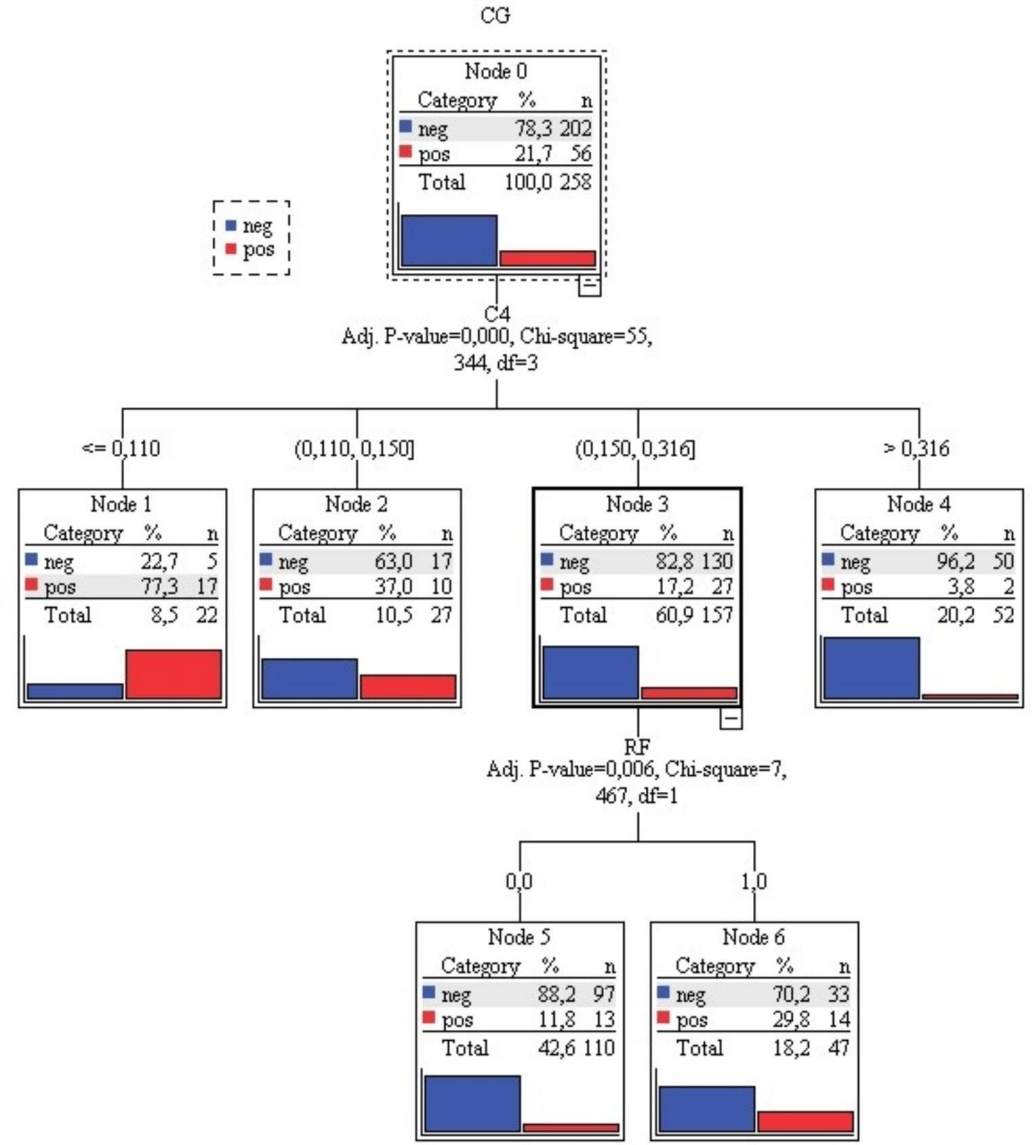
Decision tree structure (CHAID assay) for cryoglobulinemia, cut-off values of predictive immunological variables: C4 (4 groups with ranges) and RF (negative RF – 0, positive RF – 1). Legend: CG – cryoglobulins, neg – negative CG result, pos – positive CG result, RF – rheumatoid factor

The results from RF, C3c, and C4 were used to construct a CHAID decision tree model (Fig. 5) to determine how these variables best interact to explain cryoglobulinemia (qualitative positive or negative outcome).

The predictor most strongly associated with cryoglobulinemia was C4 (χ2 = 55.344 and p < 0.0001). The model divided patients into four different groups: Group I: C4 concentration < 0.110 g/L, CG were negative in 22.7% of patients, Group II: C4 concentration 0.110–0.150 g/L, CG were negative in 63.0% of patients, Group III: C4 concentration 0.150–0.316 g/L, CG were negative in 82.8% of patients and Group IV: C4 concentration > 0.316 g/L, CG were negative in 96.2% of patients. Less than 4% of patients with C4 concentration > 0.316 g/L had cryoglobulinemia. Patients in the Group III could be divided into two subgroups based on the RF result (χ2 = 7.467 and p < 0.006). In this group, only 11.8% of patients with negative RF had cryoglobulinemia, while 29.8% of patients with positive RF had cryoglobulinemia.

The overall accuracy of our CHAID decision tree model for predicting cryoglobulinemia was 82.9%. Specifically, 97.5% of patients were correctly classified as negative and only 30.4% of patients were correctly classified as positive. According to these results, only negative prediction could be further analyzed. We found that patients with a C4 value of > 0.316 g/L (Group IV) and patients with a C4 value between 0.150 and 0.316 g/L (Group III) who had a negative RF probably did not have CG (index 122.8% and 112.6%, respectively).

## Discussion

This article presents a protocol proposed by Kolopp-Sarda et al. [27] for the detection of CG, the procedure for its implementation and comparability with the current protocol, the distribution of CG in Slovenian rheumatic patients, and a decision tree analysis to search for predictors of cryoglobulinemia.

We performed evaluations to introduce the protocol of Kolopp-Sarda et al. in our routine diagnostic analyses because it is the most sensitive and specific method for confirming cryoglobulinemia. Evaluations consisted of comparison of results between FC and Kolopp-Sarda et al. protocols and verification procedures.

During the 8-month implementation period, we used FC and Kolopp-Sarda et al. protocols in CG diagnostic in parallel and found only 86% agreement. There could be several reasons for the discrepancy observed in 14.0% of the samples tested. One of the reasons could be the incubation time, as some CG require at least 7 days to precipitate. In the FC protocol, the incubation time ranged from 5 to 8 days. A shorter incubation time could lead to a false negative result for some samples in the FC method. False positive results in the FC method could also be due to an inefficient washing procedure, as proteins that have not been washed out may react with FC, leading to positive results.

We have presented here the comparison of the two protocols, however in the literature, other protocols have also been described, including measurement of total isolated immunoglobulin concentration by spectrophotometry (that this is similar to the FC protocol, because the purity of the cryoprecipitate also plays an important role in this protocol). Another assay described is the measurement of cryocrit (% volume of cryoprecipitate/total serum) after cold centrifugation, but this protocol only estimates the CG concentration. Furthermore, coprecipitating proteins could lead to a false positive result with both methods as well as with the FC protocol. Therefore, this procedure can be used for strongly positive samples, but for samples with low CG concentration, it could lead to false positive results [1, 8, 27, 30, 31].

Because of the specific characteristics of the different tests used to detect CG, we chose the protocol of Kolopp-Sarda et al., in which specific immunofixation was performed to define the types of CG, which is not possible with the other three protocols, and in which, in addition, the concentrations of IgG, IgM, and IgA were measured with nephelometry and, knowing the initial serum volume, the exact concentration of CG (IgG, IgM, and IgA) could be calculated. In addition, coprecipitation of other proteins in the Kolopp-Sarda et al. protocol does not result in false positivity because of the specific assays used in the protocol.

In addition, our study evaluated the analytical sensitivity of CG detection by immunofixation and the precision of CG detection by immunofixation and by nephelometer. The analytical sensitivity and precision of IgG, IgM, and IgA were fully in accordance with the manufacturer’s specifications and generally accepted coefficients of variation using commercially available controls. The real precision study for detection of IgG, IgM and IgA in cryoprecipitate by nephelometry was very limited due to the small number of samples with high positive CG and also due to the small volumes that remained after completion of the analyses.

Furthermore, using a protocol by Kolopp-Sarda et al., we examined the immunological characteristics of CG in terms of characterization and quantification in patients treated in the Department of Rheumatology. 21.7% (56/258) of the samples sent to our laboratory by the Department of Rheumatology for CG identification were positive. We identified 37.5% of patients with type II CG and 62.5% of patients with type III CG.

Due to differences in patient recruitment and laboratory procedures, the general distribution of CG type among different diseases is inconsistent in the literature.

In comparison, Kolopp-Sarda et al. reported 12.5% positive samples in the cohort of 13,439 patients, 9.3% of patients with type I, 47% of patients with type II, and 43.7% of patients with type III CG [30]. The difference with our study might be due to the diagnoses of the patients, because the patients included in the study by Kolopp-Sarda et al. were from different specialties: internal medicine, neurology, hepatology, nephrology, rheumatology, dermatology, pneumology/cardiology, hematology, and infectious diseases.

According to the definition of Brouet et al. [15], mixed cryoglobulinemias consist of monoclonal/polyclonal IgM with RF activity and according to Napodano et al. [26], 95% of CG consist of immune complexes with RF, however in reality, studies show a lower percentage of samples with RF activity since RF is in complex with polyclonal IgG and could only be detected with complex dissociation, which is not routinely used. In our study, only 21.4% of positive samples had RF activity. In the future, techniques for efficient resolution of immune complexes need to be introduced into routine analyses of CG to better assess the RF activity.

CHAID decision tree analysis was applied for clinically efficient evaluation of cryoglobulinemias. It is known that C4 and C3c are indicators of CG activity [21]. In our model, which included RF, C4, and C3c in serum for positive CG, C4 was the strongest predictor of cryoglobulinemia. Most importantly, our model predicted absence of CG very accurately, because patients with a C4 value > 0.316 g/L and patients with a C4 value between 0.150 and 0.316 g/L who had a negative RF were also likely to have negative CG. Similar to our findings, Stoyanov et al. showed that RF analysis together with serum protein electrophoresis has a high sensitivity for the presence of cryoglobulinemia, with a low probability of significant CG in the absence of a paraprotein in electrophoresis or detectable RF, providing a rapid and effective screening strategy [32]. The development of screening strategies would have the greatest impact on already burdened chronic patients. To our knowledge, this is the first study to use CHAID decision tree analysis to explore predictors of cryoglobulinemia. Our decision tree should be evaluated in the future in a group with a larger number of patients, including type I CG and patients with other diseases in whom CG might occur.

This study has some limitations. One is the small number of positive samples, and the second is that the cryoglobulinemic vasculitis samples were analyzed at follow-up and not at diagnosis. Also, only patients coming for consultation at rheumatologist were included in it as patient control groups.

In summary, the detection of CG in routine laboratory medicine is challenging because preanalytical factors account for most of the variability in assays. Therefore, the rigorous preanalytical phase and sensitive and specific assays for accurate CG measurement should be used in routine practice to adequately assist clinicians.
